# Prevalence of and risk factor for community-onset third-generation cephalosporin-resistant *Escherichia coli* bacteremia at a medical center in Taiwan

**DOI:** 10.1186/s12879-019-3880-z

**Published:** 2019-03-12

**Authors:** Wu-Pu Lin, Yu-Shan Huang, Jann-Tay Wang, Yee-Chun Chen, Shan-Chwen Chang

**Affiliations:** 1 Division of Infectious Diseases, Department of Internal Medicine, Taipei Hospital, Ministry of Health and Welfare, New Taipei City, Taiwan; 2 Division of Infectious Diseases, Department of Internal Medicine, National Taiwan University Hospital, Taipei City, Taiwan; 3 National Institute of Infectious Diseases and Vaccinology, National Health Research Institutes, Miaoli County, Taiwan

**Keywords:** Third-generation cephalosporin resistance, *Escherichia coli*, Bacteremia, Community-onset infection, Appropriate empirical treatment

## Abstract

**Background:**

Increased resistance to third-generation cephalosporin (3GC) is a serious concern for community-onset *Escherichia coli* infection because this resistance easily delays effective treatment. This study surveyed the current antimicrobial resistance pattern among *E. coli* isolates that cause community-onset bacteremia, with a special focus on the prevalence of and the risk factors for 3GC resistance, and determined factors for poor outcomes among patients with community-onset *E. coli* bacteremia.

**Methods:**

This retrospective study was conducted at a tertiary-care teaching hospital in Taiwan. All adult patients with community-onset *E. coli* bacteremia between January 1, 2015, and December 31, 2015 were enrolled and were divided into two groups depending on whether the *E. coli* isolate was susceptible to 3GCs. Risk factors for 3GC resistance, 14-day all-cause mortality, and length of hospital stay were analyzed.

**Results:**

The overall rate of 3GC resistance among *E. coli* isolates causing community-onset bacteremia was 19.7%, whereas it was 9.6% if only isolates causing community-acquired bacteremia were considered. Independent risk factors for 3GC-resistant community-onset *E. coli* bacteremia were hospitalization within the past 1 year (odds ratio: 2.4, 95% confidence interval: 1.6–3.7, *P* < 0.001), exposure to antibiotics within the past 15 days (2.6, 1.4–4.9, *P* = 0.002), residence in nursing home or long-term care facility (3.6, 1.0–12.3, *P* = 0.044), presence of underlying genitourinary disease (1.9, 1.2–2.9, *P* = 0.005), and presence of indwelling implantable intravenous port (2.2, 1.1–4.1, *P* = 0.021). 3GC resistance was independently associated with increased length of hospital stays (*P* < 0.001).

**Conclusion:**

In this study, the prevalence of 3GC resistance was high among both patients with community-onset and those with community-acquired *E. coli* bacteremia. 3GC resistance is a strong independent risk factor for length of hospital stay. The effectiveness of empirical antibiotic treatment would partially explain the impact of 3GC resistance, but more evidence is needed. The choice of appropriate empirical antibiotics for community-onset *E. coli* bacteremia might impact outcomes in terms of the length of hospital stay and need to be individualized according to the patient-specific risk for acquiring drug-resistant pathogens.

## Background

*Escherichia coli* is one of the leading pathogens causing community-acquired infections. In recent years, the threat of antibiotic resistance among *E. coli* and other *Enterobacteriaceae* has caused great concern [1, 2]. Moreover, a significant increase in community-acquired infections caused by extended-spectrum β-lactamase (ESBL)- or AmpC β-lactamase-producing *E. coli* strains has been observed worldwide [3, 4]. In addition to being resistant to most of the cephalosporins, these *E. coli* strains are often coresistant to fluoroquinolones and other first-line antibiotics [5]. For patients infected with these drug-resistant *E. coli*, adequate antimicrobial therapy easily gets delayed [6].

In 2010, the Clinical and Laboratory Standards Institute (CLSI) published the revised minimum inhibitory concentration interpretive criteria for *Enterobacteriaceae*. Routine ESBL reporting is no longer recommended if the new breakpoints are adopted [7]. Resistance to third-generation cephalosporins (3GCs), especially cefotaxime, has been proven to be a suitable surrogate marker for identifying ESBL- or AmpC β-lactamase-producing *E. coli* strains [8, 9]. It is now believed that antibiotic treatment can be reliably guided through antimicrobial susceptibility testing without information regarding ESBL or AmpC β-lactamase production. However, studies on bacteremia caused by 3GC-resistant *E. coli* based on the revised breakpoints remain relatively few [10, 11].

In Taiwan, a study in 2001 showed that healthcare exposure can be found in most cases with community-onset bacteremia due to drug-resistant *E. coli*, and the 3GC resistance rate was only 0.5% among *E. coli* isolates for those cases without known healthcare-associated risk factors [12]. A recent report from Taiwan Surveillance of Antimicrobial Resistance showed that the rate of 3GC-resistant *E. coli* isolates collected in outpatient clinics and emergency rooms was 8.2% in 2002 yet up to 21.1% in 2012, and was even higher (25.8%) among the elderly people [9]. However, because of insufficient clinical information in that study, it was unclear whether those drug-resistant isolates were associated with certain healthcare exposure or were truly acquired in the community settings. Therefore, the actual rate of 3GC resistance among *E. coli*-caused community-onset bacteremia without healthcare exposure history and its implication in clinical outcomes remain unclear.

In this retrospective observational study, we aimed to identify the current antimicrobial resistance pattern, especially 3GC resistance, among *E. coli* isolates causing community-onset bacteremia. Furthermore, we aimed to disclose the risk factors for 3GC resistance, 14-day all-cause mortality, and length of hospital stay. The information may guide the selection of empirical antibiotics for patients with community-onset *E. coli* bacteremia.

## Methods

### Hospital setting and study design

This retrospective study was conducted at National Taiwan University Hospital (NTUH), a 2323-bed tertiary-care teaching hospital in northern Taiwan. In 2015, NTUH received approximately 110,000 emergency visits, discharged approximately 90,000 patients, and served approximately 2,600,000 patients at outpatient clinics. All adult patients with community-onset *E. coli* bacteremia between January 1, 2015, and December 31, 2015 were enrolled. For patients with multiple episodes of *E. coli* bacteremia, only the first episode was included in the analysis. Patients were then divided into two categories, 3GC-susceptible group and 3GC-resistant group, based on whether the *E. coli* isolate was susceptible to 3GCs.

The study protocol was approved by the Institutional Review Board of NTUH (IRB No. 201707058RINB). Both oral and written informed consent were waived because of the retrospective study design and the research posing minimal risk.

### Definition

*E. coli* bacteremia was defined as at least one set of bacterial culture being positive for *E. coli*, and community-onset bacteremia was defined as the bacteremia being present at outpatient clinics or being identified within 48 h after arrival of emergency departments or after admission [4, 13]. Community-acquired bacteremia was defined as community-onset bacteremia developed in patients who had not resided in a long-term care facility, had not been hospitalized in an acute care facility, had not been treated with use of central intravenous catheters or long-term venous access devices, had not used urinary catheters, had not used other long-term percutaneous devices, had not undergone prior surgical procedures, and had not received dialysis within 1 year before the onset of bacteremia [14]. Urosepsis was defined as the primary focus of the bacteremia being of urinary origin. Persistent bacteremia was defined as obtaining at least two positive blood cultures on three days apart during the same infection episode [15].

3GC resistance was defined as the nonsusceptibility of *E. coli* isolate to either cefotaxime or ceftazidime. In our study, the empirical antibiotic treatment was considered effective if the *E. coli* isolate identified in the blood culture was susceptible to one of the antibiotics prescribed within 48 h after the blood culture was obtained. Neutropenia was defined as a white blood cell count of ≤500/μL.

### Data collection

By reviewing the medical records of the enrolled patients, we retrospectively collected data from the hospital information systems and laboratory information systems of our hospital. The basic information included demographic characteristics, underlying diseases, healthcare facility utilization, and the presence of any indwelling mucosal or intravenous catheter. Furthermore, the following comorbid conditions were identified: cardiovascular disease, respiratory disease, gastrointestinal disease, hepatobiliary disease, genitourinary disease, diabetes mellitus, and malignancy. Charlson comorbidity index for each patient was calculated. Moreover, we checked the electronic medical records to note whether the patient received any antimicrobial agents within the previous 15 days or any recent surgery before the onset of bacteremia. The primary focus of infection was determined based on the clinical presentation and final diagnosis made by the primary care clinician. Furthermore, the presence of shock episode when the patient was presented was recorded.

Laboratory reports at the presentation of *E. coli* bacteremia were reviewed. Blood culture results, including the date of sampling and the antimicrobial susceptibility testing, were collected. The information regarding empirical antibiotic treatment was recorded.

### Microbiologic studies

Blood cultures were processed at a clinical microbiology laboratory. Species identification and antimicrobial susceptibility testing were performed using the VITEK II system (bioMérieux, Durham, NC, USA) following the guidelines of the manufacturer and CLSI [7]. The susceptibility to tigecycline was interpreted using breakpoints proposed by the European Committee on Antimicrobial Susceptibility Testing (EUCAST) (http://www.eucast.org/clinical_breakpoints/).

### Statistical analysis

STATA software package version 14.0 (StataCorp, College Station, TX, USA) was used to perform the analysis. Continuous variables were compared using the Student *t* test if normally distributed or the Mann–Whitney *U* test if nonnormally distributed. Categorical variables were compared using the Chi-square test or Fisher’s exact test and were reported as proportions.

Risk factors for 3GC resistance and 14-day all-cause mortality were analyzed using logistic regression, whereas risk factors for the length of hospital stay were analyzed using linear regression. The analysis for the latter was limited to the patients who did not die during hospitalization. Factors with a *P* value of < 0.1 in the univariate analysis or those with biological meanings were candidates for multivariate analysis using a backward elimination method. A *P* value of < 0.05 was considered statistically significant.

## Results

**Fig. 1:**
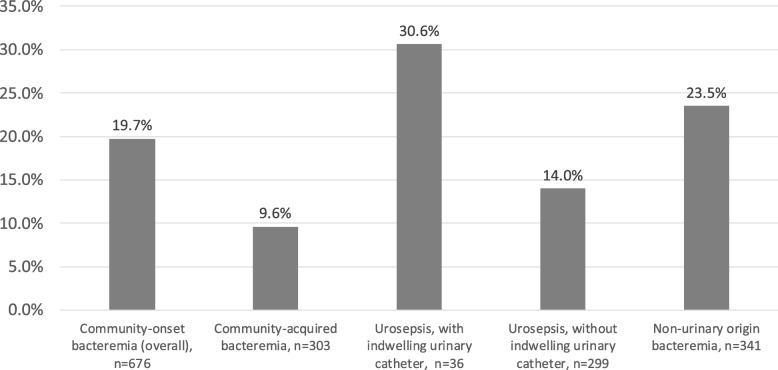
Resistance rate of third-generation cephalosporin among different groups of patients with *Escherichia coli* bacteremia

In total, 676 adult patients with community-onset *E. coli* bacteremia were enrolled. Of these, the *E. coli* isolates of 543 patients were susceptible to 3GCs and those of 133 patients were resistant to 3GCs. The overall rate of 3GC resistance was 19.7% (Fig. 1). The bacteremia of 303 patients were considered as community-acquired bacteremia, and the 3GC resistance rate was lower among these patients (29 of 303, 9.6%). Among patients with urosepsis, the 3GC resistance rate was 15.8% (53 of 335). The rate was higher among patients with urosepsis who had indwelling urinary catheter (11 of 36, 30.6%).

Patient characteristics are shown in Table 1. Compared with patients in the 3GC-susceptible group, those in the 3GC-resistant group showed less female predominance, had a higher proportion of medical facility utilization or indwelling catheter use, had a higher Charlson comorbidity index, had a higher proportion of patients with a history of hospitalization or antibiotic use, and had a lower proportion of patients with bacteremia of urinary origin.

**Table 1:** Characteristics of patients with community-onset *Escherichia coli* bacteremia

Characteristic	Overall (*n* = 676)	3GC-susceptible (*n* = 543)	3GC-resistant (*n* = 133)
Age, years, mean ± SD	67.1 ± 15.5	66.8 ± 15.5	68.0 ± 15.2
Sex, female^a^	419 (62.0)	347 (63.9)	72 (54.1)
Hospitalization within the past 1 year^a^	305 (45.1)	215 (39.6)	90 (67.7)
Exposure to antibiotics within the past 15 days^a^	55 (8.2)	32 (5.9)	23 (17.3)
Medical facility utilization^a^:			
OPD for hypertension/ diabetes mellitus	473 (70.0)	373 (68.7)	100 (75.2)
OPD for chemotherapy	95 (14.0)	65 (12.0)	30 (22.6)
Hemodialysis or peritoneal dialysis	24 (3.6)	14 (2.6)	10 (7.5)
Nursing home/Long-term care facility	12 (1.8)	6 (1.1)	6 (4.5)
Charlson comorbidity index, median (range)	2 (0–13)	2 (0–11)	3 (0–13)
Indwelling mucosal catheter^a^	129 (19.1)	84 (15.5)	45 (33.8)
Indwelling intravenous catheter^a^	100 (14.8)	64 (11.8)	36 (27.1)
Primary focus^a^:			
Primary bacteremia	75 (11.1)	54 (9.9)	21 (15.8)
Urinary tract	335 (49.6)	282 (51.9)	53 (39.9)
Intra-abdomen	182 (26.9)	143 (26.3)	39 (29.3)
Pulmonary	7 (1.0)	2 (0.4)	5 (3.8)
Others	23 (3.3)	17 (3.1)	5 (3.8)
Unknown	55 (8.1)	45 (8.3)	10 (7.5)
Neutropenia^a^	25 (3.7)	18 (3.3)	7 (5.3)
Hospital days, median (range)	15 (0–159)	14 (0–157)	18 (0–159)

^a^ Data are presented as no. (%) of patients

Abbreviation: *3GC* third-generation cephalosporin, *OPD* outpatient department

The percentage of patients who received effective empirical antibiotic treatment was 93% in the 3GC-susceptible group and 46% in the 3GC-resistant group (*P* < 0.001) (Table 2). Compared with patients in the 3GC-susceptible group, those in the 3GC-resistant group were more likely to die within 48 h (7% vs. 2%, *P* = 0.011), had a higher 14-day all-cause mortality rate (16% vs. 8%, *P* = 0.005), and had longer hospital days after excluding those who died during hospitalization (median, 18 days vs. 14 days, *P* < 0.001).

**Table 2:** Percentage of effective empirical antibiotics and the outcomes for patients with community-onset *Escherichia coli* bacteremia

	3GC-susceptible (*n* = 543)	3GC-resistant (n = 133)	*P* value
Effective empirical antibiotic treatment^a^	503 (92.6)	61 (45.9)	< 0.001
Death within 48 hours^a^	13 (2.4)	9 (6.8)	0.011
Persistent bacteremia^a^	72 (13.3)	18 (13.5)	0.963
Death within 14 days^a^	43 (7.9)	21 (15.8)	0.005
Length of hospital stay, median (range)^b^	14 (3–157)	18 (6–159)	< 0.001^c^

^a^Data are presented as no. (%) of patients

^b^Excluding patients who died during hospitalization (only 396 patients in the 3GC-susceptible group and 100 in the 3GC-resistant group were analyzed)

^c^Using Mann–Whitney *U* test

Abbreviation: *3GC* third-generation cephalosporin

We then compared patients who received effective empirical antibiotic treatment with those who did not. As shown in Table 3, no significant difference was observed between the two groups in terms of Charlson comorbidity index or shock percentage on presentation. Furthermore, the 14-day all-cause mortality rate was similar. However, the median hospital days were significantly longer among patients who did not receive effective empirical antibiotic treatment (17 days vs. 14 days, *P* = 0.005). Among patients with community-acquired bacteremia or urosepsis, the median days of hospitalization were also significantly longer among those who did not receive effective empirical antibiotic treatment compared with those who did.

**Table 3:** Disease severity and outcomes for patients by whether they received effective empirical antibiotic treatment

Disease severity / Outcomes	Community-onset bacteremia			Community-acquired bacteremia			Urosepsis		
	Effective Tx (*n* = 564)	Ineffective Tx (*n* = 112)	*P* value	Effective Tx (*n* = 267)	Ineffective Tx (*n* = 36)	*P* value	Effective Tx (*n* = 281)	Ineffective Tx (n = 54)	*P* value
Charlson comorbidity index, median (range)	2 (0–11)	2 (0–13)	0.587^b^	1 (0–10)	1 (0–6)	0.294^b^	2 (0–11)	2 (0–11)	0.413^b^
Shock upon presentation (%)	94 (16.7)	13 (11.6)	0.180	19 (7.1)	1 (2.8)	0.487^c^	35 (12.5)	3 (5.6)	0.166^c^
Death within 14 days (%)	54 (9.6)	10 (8.9)	0.831	6(2.3)	0(0)	0.364	13(4.6)	2(3.7)	0.764
Hospital stay, median days (range)^a^	14 (3–159)	17 (6–80)	0.005^b^	12 (3–44)	16 (6–52)	0.002^b^	14 (3–159)	15 (7–80)	0.039^b^

^a^Excluding patients who died during hospitalization (Among patients with community-onset bacteremia, only 413 patients who received effective empirical antibiotic treatment and 83 patients who received ineffective empirical antibiotic treatment were analyzed. In community-acquired bacteremia group, only 191 patients who received effective empirical antibiotic treatment and 28 patients who received ineffective empirical antibiotic treatment were analyzed, whereas in urosepsis group, only 223 patients who received effective empirical antibiotics and 41 patients who received ineffective empirical antibiotic treatment were analyzed)

^b^Compared by Mann–Whitney *U* test

^c^Compared by Fisher’s exact test

Abbreviation: *Tx* empirical antibiotics treatment

### Risk factors for 3GC resistance

In univariate analysis using logistic regression, the following risk factors were statistically significant: hospitalization within the past 1 year; exposure to antibiotics within the past 15 days; fluoroquinolone use within the past 15 days; regular outpatient follow-up for chemotherapy; receipt of hemodialysis or peritoneal dialysis; residence in nursing home or long-term care facility; Charlson comorbidity index ≥2; presence of a urinary catheter or implantable port; presence of underlying gastrointestinal or genitourinary disorder, diabetes mellitus, or malignancy; nonurinary focus of infection; and shock upon presentation (Table 4). In multivariate analysis, hospitalization within the past 1 year (odds ratio [OR]: 2.4; 95% confidence interval [CI]: 1.6–3.7; *P* < 0.001), exposure to antibiotics within the past 15 days (OR: 2.6; 95% CI: 1.4–4.9; *P* = 0.002), residence in nursing home or long-term care facility (OR: 3.6; 95% CI: 1.0–12.3; *P* = 0.044), underlying genitourinary disease (OR: 1.9; 95% CI: 1.2–2.9; *P* = 0.005), and the presence of implantable intravenous port (OR: 2.2; 95% CI: 1.1–4.1; *P* = 0.021) were identified as independent risk factors for community-onset 3GC-resistant *E. coli* bacteremia (Table 5). Subgroup analysis showed hospitalization within 1 year (OR: 2.3; 95% CI: 1.2–4.3; *P* = 0.011), residence in nursing home or long-term care facility (OR: 5.0; 95% CI: 1.1–22.2; *P* = 0.034), and underlying genitourinary disease (OR: 3.2; 95% CI: 1.7–6.0; *P* < 0.001) as independent risk factors for 3GC resistance among patients with community-onset *E. coli* bacteremia due to urosepsis, whereas no single independent risk factor for 3GC resistance could be identified among patients with community-acquired *E. coli* bacteremia. For those patients with community-onset *E. coli* bacteremia who didn’t meet the definition of community-acquired bacteremia, the independent risk factors for 3GC resistance included exposure to antibiotics within the past 15 days (OR: 3.0; 95% CI: 1.5–5.7; *P* = 0.001), underlying genitourinary disease (OR: 2.2; 95% CI: 1.3–3.7; *P* = 0.002), and nonurinary focus of infection (OR: 1.8; 95% CI: 1.1–3.1; *P* = 0.018).

**Table 4:** Univariate analysis of risk factors for community-onset 3GC-resistant *Escherichia coli* bacteremia

Risk factor	3GC susceptible(n = 543)	3GC resistant (*n* = 133)	OR (95% CI)	*P v*alue
Age ≥ 65 years old	313 (57.6)	82 (61.7)	1.2 (0.8–1.7)	0.400
Hospitalization within the past 1 year	215 (39.6)	90 (67.7)	3.2 (2.1–4.8)	< 0.001
Exposure to antibiotics within the past 15 days	32 (5.9)	23 (17.3)	3.3 (1.9–5.9)	< 0.001
Prior cephalosporin use within the past 15 days	23 (4.2)	11 (8.3)	2.0 (1.0–4.3)	0.061
Prior 3GC use within the past 15 days	3 (0.6)	3 (2.3)	4.2 (0.8–20.8)	0.083
Prior fluoroquinolone use within the past 15 days	1 (0.2)	4 (3.0)	16.8(1.9–151.6)	0.012
Neutropenia	18 (3.3)	7 (5.3)	1.6 (0.7–4.0)	0.290
Medical facility utilization:				
OPD for hypertension/ diabetes mellitus	373 (68.7)	100 (75.2)	1.4 (0.9–2.1)	0.144
OPD for chemotherapy	65 (12.0)	30 (22.6)	2.1 (1.3–3.5)	0.002
Hemodialysis or peritoneal dialysis	14 (2.6)	10 (7.5)	3.1 (1.3–7.1)	0.008
Nursing home/Long-term care facility	6 (1.1)	6 (4.5)	4.2 (1.3–13.3)	0.014
Charlson comorbidity index ≥2	357 (65.8)	104 (78.2)	1.9 (1.2–2.9)	0.006
Indwelling mucosal catheter:				
Urinary catheter	41 (7.6)	22 (16.5)	2.4 (1.4–4.2)	0.002
Tracheostomy/endotracheal tube	4 (0.7)	2 (1.5)	2.1 (0.4–11.4)	0.408
Nasogastric tube	43 (7.9)	14 (10.5)	1.4 (0.7–2.6)	0.334
Indwelling intravenous catheter:				
Central venous catheter	24 (4.4)	10 (7.5)	1.8 (0.8–3.8)	0.147
Implantable port	31 (5.7)	19 (14.3)	2.8 (1.5–5.0)	0.001
Underlying disease:				
Cardiovascular	283 (52.1)	71 (53.4)	1.1 (0.7–1.5)	0.793
Respiratory	58 (10.7)	18 (13.5)	1.3 (0.7–2.3)	0.352
Gastrointestinal	89 (16.4)	30 (22.6)	1.5 (0.9–2.4)	0.096
Hepatobiliary	135 (24.9)	36 (27.1)	1.1 (0.7–1.7)	0.600
Genitourinary	112 (20.6)	46 (34.6)	2.0 (1.3–3.1)	0.001
Diabetes mellitus	165 (30.4)	53 (39.9)	1.5 (1.0–2.2)	0.037
Malignancy	181 (33.3)	70 (52.6)	2.2 (1.5–3.3)	< 0.001
Recent surgery	10 (1.8)	6 (4.5)	2.5 (0.9–7.1)	0.079
Nonurinary focus of infection	261 (48.1)	80 (60.2)	1.6 (1.1–2.4)	0.013
Shock upon presentation	78(14.4)	29(21.8)	1.7 (1.0–2.7)	0.037

Abbreviations: *3GC* third-generation cephalosporin, *OR* odds ratio, *CI* confidence interval, *OPD* outpatient department

**Table 5:** Independent risk factors for community-onset 3GC-resistant *Escherichia coli* bacteremia

Risk factor	Adjusted OR (95% CI)	*P v*alue
Hospitalization within the past 1 year	2.4 (1.6–3.7)	< 0.001
Exposure to antibiotics within the past 15 days	2.6 (1.4–4.9)	0.002
Residence of nursing home / long-term care facility	3.6 (1.0–12.3)	0.044
Underlying genitourinary disease	1.9 (1.2–2.9)	0.005
Indwelling implantable intravenous port	2.2 (1.1–4.1)	0.021

Abbreviations: *OR* odds ratio, *CI* confidence interval

### Risk factors for 14-day all-cause mortality

Statistically significant factors in univariate analysis using logistic regression for 14-day all-cause mortality included female sex; neutropenia; receipt of hemodialysis or peritoneal dialysis; Charlson comorbidity index ≥2; presence of a urinary catheter, central venous catheter, or implantable intravenous port; underlying hepatobiliary disorder or malignancy; nonurinary focus of infection; shock upon presentation; 3GC resistance; and hospitalization within the past 1 year. Multivariate analysis showed that underlying malignancy (OR: 4.6, 95% CI: 2.3–9.3; *P* < 0.001), nonurinary focus of infection (OR: 2.8; 95% CI: 1.3–5.8; *P* = 0.006), and shock upon presentation (OR: 38.8; 95% CI: 19.0–79.1; *P* < 0.001) were independent risk factors for 14-day all-cause mortality in our patients with community-onset *E. coli* bacteremia (Table 6). Subgroup analysis showed the presence of an implantable intravenous port (OR: 12.6; 95% CI: 1.4–113.3; *P* = 0.024), underlying malignancy (OR: 4.8, 95% CI: 1.3–17.6; *P* = 0.020), shock upon presentation (OR: 17.1; 95% CI: 4.7–61.8; *P* < 0.001), and 3GC resistance (OR: 4.7; 95% CI: 1.3–17.2; *P* = 0.020) to be independently associated with increased 14-day all-cause mortality among patients with community-onset *E. coli* bacteremia due to urosepsis, whereas only shock upon presentation (OR: 24.8; 95% CI: 3.9–158.5; *P* = 0.001) was independently associated with increased 14-day all-cause mortality among patients with community-acquired *E. coli* bacteremia.

**Table 6:** Independent risk factors for 14-day all-cause mortality among patients with community-onset *Escherichia coli* bacteremia

Risk factor	Adjusted OR (95% CI)	*P* Value
Underlying malignancy	4.6 (2.3–9.3)	< 0.001
Nonurinary focus of infection	2.8 (1.3–5.8)	0.006
Shock upon presentation	38.8 (19.0–79.1)	< 0.001

Abbreviations: *OR* odds ratio, *CI* confidence interval

### Risk factors for length of hospital stay

In univariate linear regression analysis, the following factors were associated with increased length of hospital stay: hospitalization within the past 1 year; receipt of hemodialysis or peritoneal dialysis; Charlson comorbidity index ≥2; presence of a urinary catheter, nasogastric tube, central venous catheter, or implantable intravenous port; underlying genitourinary disorder, diabetes mellitus, or malignancy; nonurinary focus of infection; shock upon presentation; 3GC resistance; and ineffective empirical antibiotic treatment. After adjustments in the multivariate linear regression analysis, only hospitalization within the past 1 year (*P* < 0.001), presence of a indwelling central venous catheter (*P* = 0.001), receipt of hemodialysis or peritoneal dialysis (*P* = 0.020), presence of a indwelling nasogastric tube (*P* = 0.004), nonurinary focus of infection (*P* = 0.033), and 3GC resistance (*P* < 0.001) were independently associated with increased length of hospital stay in our patients with community-onset *E. coli* bacteremia (Table 7). Furthermore, 3GC resistance was significantly associated with increased length of hospital stay among both patients with community-onset *E. coli* bacteremia due to urosepsis (*P* < 0.001) and those with community-acquired *E. coli* bacteremia (*P* < 0.001).

**Table 7:** Independent risk factors for length of hospital stay among patients with community-onset *Escherichia coli* bacteremia^a^

Risk factor	Parameter (estimate in days)	95% CI	*P* value
Hospitalization within the past 1 year	5.3	2.6–7.9	< 0.001
Indwelling central venous catheter	10.8	4.5–17.0	0.001
Hemodialysis or peritoneal dialysis	9.4	1.5–17.3	0.020
Indwelling nasogastric tube use	6.8	2.2–11.4	0.004
Nonurinary focus of infection	2.8	0.2–5.5	0.033
3GC resistance	6.8	3.5–10.0	< 0.001

^a^Excluding patients who died during hospitalization (only 496 patients were analyzed)

Abbreviations: *CI* confidence interval

### Drug susceptibilities

Drug susceptibilities of *E. coli* isolates among the different groups are shown in Table 8. The overall susceptibility rates to third- and fourth-generation cephalosporins were both 80% and those to ciprofloxacin and levofloxacin were both 81%. For 3GC-resistant strains, the overall susceptibility rate to ciprofloxacin was approximately 50%, whereas the susceptibility rates to piperacillin/tazobactam and ertapenem were high (87% and 98%, respectively). The overall drug susceptibility tended to be high among isolates causing urosepsis or community-acquired bacteremia. For patients with community-acquired *E. coli* bacteremia, the susceptibility rates to both 3GC and fluoroquinolone were approaching 90%.

**Table 8:** Drug susceptibilities (%) of *Escherichia coli* isolates among different groups

Antibiotics	Community-onset bacteremia			Urosepsis		Community-acquired bacteremia	
	Overall (*n* = 676)	3GC S (n = 543)	3GC R (n = 133)	Overall (*n* = 335)	3GC S (*n* = 282)	Overall (*n* = 303)	3GC S (*n* = 274)
Amikacin	100.0	100.0	100.0	100.0	100.0	100.0	100.0
Ceftazidime	80.2	99.6	0.8	83.9	99.3	90.4	100.0
Ciprofloxacin	81.3	88.2	53.0	82.7	89.4	87.5	90.1
Cefmetazole	93.6	99.8	67.9	96.1	99.6	96.7	99.6
Cefotaxime	80.3	100.0	0.0	84.2	100.0	90.4	100.0
Cefazolin	73.8	91.9	0.0	76.4	90.8	83.8	92.7
Ertapenem	99.3	99.6	97.7	99.4	99.3	100.0	100.0
Cefepime	80.3	99.6	1.5	83.6	99.3	90.4	100.0
Gentamycin	81.4	86.9	58.6	80.0	84.0	87.1	88.7
Imipenem	99.7	99.6	100.0	99.4	99.3	100.0	100.0
Levofloxacin	81.2	88.2	52.6	82.4	89.4	87.1	90.1
Meropenem	99.7	99.6	100.0	99.4	99.3	100.0	100.0
Ampicillin/Sulbactam	44.1	52.3	10.5	42.7	48.2	51.1	55.1
Trimethoprim/Sulfamethoxazole	54.4	59.3	34.6	51.6	55.7	59.7	61.7
Tigecycline^a^	100.0	100.0	100.0	100.0	100.0	100.0	100.0
Piperacillin/Tazobactam	95.0	97.0	86.5	95.5	95.7	96.7	97.4

^a^Drug susceptibility to tigecycline was not tested on all isolates. Overall the results were only available on 460 isolates, among which 372 isolates are susceptible to 3GC. In urosepsis group, the results of drug susceptibility to tigecycline were only available on 209 isolates, among which 179 isolates are susceptible to 3GC. In community-acquired bacteremia group, the results of drug susceptibility to tigecycline were only available on 209 isolates, among which 187 isolates are susceptible to 3GC

Abbreviations: *3G*, third-generation cephalosporin, *S* susceptible, *R* resistant

## Discussion

Because *E. coli* is one of the commonest pathogens in community setting, the increasing rate of drug resistance among these pathogens is a great concern worldwide. In the present study, the overall 3GC resistance rate among *E. coli* isolates causing community-onset bacteremia was 19.7%, whereas the 3GC resistance rate among isolates causing community-acquired bacteremia was 9.6%. Independent risk factors for community-onset 3GC-resistant *E. coli* bacteremia included hospitalization within the past 1 year, exposure to antibiotics within the past 15 days, residence in nursing home or long-term care facility, underlying genitourinary disease, and the presence of an implantable intravenous port. Underlying malignancy, nonurinary focus of infection, and shock upon presentation were the only risk factors independently associated with 14-day all-cause mortality. Conversely, independent risk factors for length of hospital stay among patients with community-onset *E. coli* bacteremia included hospitalization within the past 1 year, the presence of a central venous catheter, receipt of hemodialysis or peritoneal dialysis, the presence of a nasogastric tube, nonurinary focus of infection, and 3GC resistance.

The overall 3GC resistance rate in this study (19.7%) was similar to that reported in 2012 from a multicenter study in Taiwan (21.1%) [9]. However, studies during a similar period in England, focusing on community-onset *E. coli* bacteremia nationwide, and in the central region of France, focusing on overall *E. coli* bacteremia, have reported much lower 3GC resistance rates (9.3 and 8.0%, respectively) [16, 17]. The difference might be partially explained by the proportion of patients with healthcare exposure, as in our study, only 45% (303 of 676) of the patients had community-acquired bacteremia, who, by definition, are devoid of known healthcare exposure.

The 3GC resistance rate regarding community-acquired bacteremia revealed by our study was also deserved concern. A previous study conducted in our hospital between 2001 and 2002 revealed that the 3GC resistance rate among *E. coli* isolates causing community-acquired bacteremia was 0.5%, based on 2009 CLSI criteria [12]. Using the same (2009) criteria, the 3GC resistance rate among those *E. coli* isolates causing community-acquired bacteremia in the present study was 4.9%, which was significantly higher. Furthermore, one multicenter study in China showed that the proportion of ESBL-producing strain among community-acquired *E. coli* bacteremia was up to 51% [18]. Considering the frequent traveling and business activities between Taiwan and mainland China, the prevalence of drug-resistant *E. coli* strain in Taiwan is likely to continue to increase. Other studies have shown that the prevalence of fecal carriage of ESBL-producing *E. coli* could be as high as 30% among healthy individuals in communities, indicating that these drug-resistant *E. coli* strains may have spread in communities worldwide and are able to infect patients who even have no known healthcare exposure [19–21].

The risk factors for 3GC resistance identified in our present study were similar to those identified for ESBL production in previous studies, and they basically reflect the accumulated healthcare exposure [4, 6, 13, 18, 22–25]. Nursing home as well as other long-term care facilities are thought to be a potential reservoir for multidrug-resistant pathogens, and several studies have already shown a high carriage rate of ESBL-producing *E. coli* among facility residents [26, 27]. Whether 3GC-resistant *E. coli* possibly spread in nursing homes and long-term facilities in Taiwan needs further investigation.

Whether appropriate empirical antibiotic treatment for ESBL-producing *E. coli* bacteremia decreases mortality has been debated, and studies have shown mixed results [28, 29]. Some studies have claimed that the choice of empirical antibiotics was not associated with the prognosis if the therapy was later adjusted appropriately according to susceptibility results [30–33], whereas others have argued that appropriate empirical antibiotic treatment is independently associated with more favorable outcomes [34]. Similar discussions regarding 3GC-resistant *E. coli* bacteremia are few; our study found that the effectiveness of the empirical antibiotic treatment does not have a significant impact on the mortality rate, which is similar to the findings of two other studies [10, 35]. For community-onset *E. coli* bacteremia, mortality is usually quite low (< 10% in our study), and the severity tends to be lower. Most of the time, it is not too late for the patient to receive adequate antibiotic treatment after the drug susceptibility is known, although the initial antibiotic therapy is ineffective. In this scenario, the impact of appropriate empirical antibiotic treatment on mortality rate would be conceivably limited. Therefore, it is reasonable that overall, only the underlying condition and disease severity had a significant impact on 14-day all-cause mortality in our study.

In this study, risk factors that were associated with increased length of hospital stays reflected the underlying conditions of patients, as patients with more comorbidities would have more complications in a similar bacteremic episode and thus would be admitted longer. The finding that 3GC resistance was independently associated with increased length of hospital stay is, however, intriguing. de Kraker and colleagues showed a similar result, but no explanation was provided about the phenomenon [36]. In our study, a significantly higher proportion of patients with 3GC-susceptible *E. coli* bacteremia received effective empirical antibiotic treatment than those with 3GC-resistant *E. coli* bacteremia (93% vs. 46%, *P* < 0.001). Moreover, patients who received effective empirical antibiotic treatment had a shorter length of hospital stay than those who did not (14 days vs. 17 days, *P* = 0.005). This might imply that the impact of 3GC resistance on the duration of hospital stay could be at least partially explained by the difference in the proportion of effective empirical antibiotic use in addition to medical facility utilization and Charlson comorbidity index, though in our study ineffective empirical antibiotic treatment failed to be a statistically significant risk factor for length of hospital stay. More studies would be needed to clarify the impact of the empirical antibiotic itself on the outcome of the patients with community-onset *E. coli* bacteremia. Whether *E. coli* isolates with 3GC resistance per se increases hospitalization duration through some other mechanisms deserves further investigation.

To choose appropriate empirical antibiotic agents, it is essential to estimate the risk of a patient acquiring drug-resistant infection. Our study identified several independent risk factors for 3GC-resistant *E. coli* bacteremia, using which clinicians could stratify their patients and predict the risk. If the risk of acquiring 3GC-resistant *E. coli* is high, then 3GC would not be reliable empirical agents. Fluoroquinolone, another class of common empirical antibiotic agent to treat infections in a community setting, should also be prescribed with caution as the overall resistance rate of *E. coli* to this agent was high and that of 3GC-resistant strains was even higher (19% and 47%, respectively, in this study). In other words, as drug resistance has become an issue for community-onset infection, the choice of empirical agents should be individualized, and the local antimicrobial resistance pattern of the possible causative pathogens should be taken into consideration.

This study has several strengths: It was one of the few studies that focused on 3GC resistance of *E. coli* isolates that cause community-onset bacteremia, and the sample size was relatively large. Only patients with confirmed *E. coli* bacteremia were enrolled, and each *E. coli* isolate caused active infection and not just colonization. All cases were enrolled within a single year, 2015, which minimized background variation and provided updated antimicrobial resistance pattern. We collected detailed information regarding patients’ healthcare-associated exposure and clinical presentation; thus, subgroup analysis could be performed. Finally, we performed further analysis focusing on community-acquired bacteremia and urosepsis, two representative entities among community-onset severe infections, and the results would help clinicians better evaluate their patients in a community setting.

One of the limitations of this study is the lack of data on the distribution of drug-resistant mechanism. For 3GC-resistant *E. coli*, both ESBL and AmpC β-lactamase are commonly responsible enzymes causing resistance, and the risk factors for acquiring *E. coli* isolates with different enzymes are distinct. Nevertheless, it reflects the clinical scenario that the drug-resistant mechanisms of 3GC-resistant *E. coli* are not available to clinicians on a routine basis. The fact that no single risk factor for 3GC resistance was identified in our study among patients with community-acquired bacteremia may indicate that some nonhealthcare-associated risk factors are still missing. For example, a previous study pointed out that the living environment may play a role in the subsistence and transmission of ESBL-producing *E. coli* in a community [37]. The identification of nonhealthcare-associated risk factors would help to explain the increasing drug resistance rate among isolates causing community-acquired infection. Finally, patients enrolled in our study may have more than average healthcare-associated exposure because our hospital is a medical and referral center. The difference in source population should be taken into consideration when the result of this study is applied.

## Conclusions

Our study showed that the prevalence of 3GC resistance was 19.7% among patients with community-onset *E. coli* bacteremia, whereas it was 9.6% among patients with community-acquired *E. coli* bacteremia. Overall, 3GC resistance was not associated with 14-day all-cause mortality but was significantly associated with increased length of hospital stay. The effectiveness of empirical antibiotic treatment might partially explain the impact of 3GC resistance, but more evidence is needed. Whether 3GC resistance could prolong hospital stay through other mechanisms deserves further investigation. In an era when antibiotic resistance is a common issue, the choice of appropriate empirical antibiotic agents for severe infections in a community setting is challenging and needs to be individualized according to the patients’ risk of acquiring drug-resistant pathogens.

## Abbreviations

3GC: third-generation cephalosporin
CLSI: clinical and laboratory standards institute
ESBL: extended-spectrum β-lactamase
EUCAST: European committee on antimicrobial susceptibility testing
NTUH: National Taiwan University Hospital

## References

[1] Partridge SR (2015). Resistance mechanisms in Enterobacteriaceae. Pathology..

[2] Sidjabat HE, Paterson DL (2015). Multidrug-resistant Escherichia coli in Asia: epidemiology and management. Expert Rev Anti-Infect Ther.

[3] Chong Y, Shimoda S, Yakushiji H, Ito Y, Miyamoto T, Kamimura T (2013). Community spread of extended-spectrum beta-lactamase-producing Escherichia coli, Klebsiella pneumoniae and Proteus mirabilis: a long-term study in Japan. J Med Microbiol.

[4] Rodriguez-Bano J, Picon E, Gijon P, Hernandez JR, Ruiz M, Pena C (2010). Community-onset bacteremia due to extended-spectrum beta-lactamase-producing Escherichia coli: risk factors and prognosis. Clinical infectious diseases : an official publication of the Infectious Diseases Society of America..

[5] Durmaz S, Percin D, Ercal BD (2015). Molecular epidemiology of quinolon resistant strains of extended spectrum beta-lactamase producing Escherichia coli. Pak J Med Sci.

[6] Nguyen ML, Toye B, Kanji S, Zvonar R (2015). Risk factors for and outcomes of bacteremia caused by extended-Spectrum ss-lactamase-producing Escherichia coli and Klebsiella species at a Canadian tertiary care hospital. Can J Hosp Pharm.

[7] CLSI (2015). Performance standards for antimicrobial susceptibility testing: 25th informational supplement M100-S25.

[8] Rossolini GM, D'Andrea MM, Mugnaioli C (2008). The spread of CTX-M-type extended-spectrum beta-lactamases. Clinical microbiology and infection : the official publication of the European Society of Clinical Microbiology and Infectious Diseases..

[9] Wang JT, Chang SC, Chang FY, Fung CP, Chuang YC, Chen YS (2015). Antimicrobial non-susceptibility of Escherichia coli from outpatients and patients visiting emergency rooms in Taiwan. PLoS One.

[10] Lee S, Han SW, Kim KW, Song DY, Kwon KT (2014). Third-generation cephalosporin resistance of community-onset Escherichia coli and Klebsiella pneumoniae bacteremia in a secondary hospital. The Korean journal of internal medicine.

[11] Bidell MR, Palchak M, Mohr J, Lodise TP (2016). Fluoroquinolone and third-generation-cephalosporin resistance among hospitalized patients with urinary tract infections due to Escherichia coli: do rates vary by hospital characteristics and geographic region?. Antimicrob Agents Chemother.

[12] Sun HY, Chen SY, Chang SC, Pan SC, Su CP, Chen YC (2006). Community-onset Escherichia coli and Klebsiella pneumoniae bacteremia: influence of health care exposure on antimicrobial susceptibility. Diagn Microbiol Infect Dis.

[13] Park YS, Bae IK, Kim J, Jeong SH, Hwang SS, Seo YH (2014). Risk factors and molecular epidemiology of community-onset extended-spectrum beta-lactamase-producing Escherichia coli bacteremia. Yonsei Med J.

[14] Wang JL, Chen SY, Wang JT, Wu GH, Chiang WC, Hsueh PR (2008). Comparison of both clinical features and mortality risk associated with bacteremia due to community-acquired methicillin-resistant Staphylococcus aureus and methicillin-susceptible S. Aureus. Clinical infectious diseases : an official publication of the Infectious Diseases Society of America.

[15] Canzoneri CN, Akhavan BJ, Tosur Z, Andrade PEA, Aisenberg GM. Follow-up Blood Cultures in Gram-Negative Bacteremia: Are They Needed?. Clin Infect Dis. 2017;65(11):1776–9. 10.1093/cid/cix648 29020307

[16] Bou-Antoun S, Davies J, Guy R, Johnson AP, Sheridan EA, Hope RJ. Descriptive epidemiology of *Escherichia coli* bacteraemia in England, April 2012 to March 2014. Euro Surveill. 2016;21(35). 10.2807/1560-7917.ES.2016.21.35.30329 PMC5015457 27608263

[17] van der Mee-Marquet NL, Blanc DS, Gbaguidi-Haore H, Dos Santos BS, Viboud Q, Bertrand X (2015). Marked increase in incidence for bloodstream infections due to Escherichia coli, a side effect of previous antibiotic therapy in the elderly. Front Microbiol.

[18] Quan J, Zhao D, Liu L, Chen Y, Zhou J, Jiang Y (2017). High prevalence of ESBL-producing Escherichia coli and Klebsiella pneumoniae in community-onset bloodstream infections in China. J Antimicrob Chemother.

[19] Ni Q, Tian Y, Zhang L, Jiang C, Dong D, Li Z (2016). Prevalence and quinolone resistance of fecal carriage of extended-spectrum beta-lactamase-producing Escherichia coli in 6 communities and 2 physical examination center populations in Shanghai. China Diagn Microbiol Infect Dis.

[20] Mathai D, Kumar VA, Paul B, Sugumar M, John KR, Manoharan A (2015). Fecal carriage rates of extended-spectrum beta-lactamase-producing *Escherichia coli* among antibiotic naive healthy human volunteers. Microbial drug resistance (Larchmont, NY).

[21] Ahmed SF, Ali MM, Mohamed ZK, Moussa TA, Klena JD (2014). Fecal carriage of extended-spectrum beta-lactamases and AmpC-producing Escherichia coli in a Libyan community. Ann Clin Microbiol Antimicrob.

[22] Park SY, Kang CI, Joo EJ, Ha YE, Wi YM, Chung DR (2012). Risk factors for multidrug resistance in nosocomial bacteremia caused by extended-spectrum beta-lactamase-producing *Escherichia coli* and *Klebsiella pneumoniae*. Microbial drug resistance (Larchmont, NY).

[23] Serefhanoglu K, Turan H, Timurkaynak FE, Arslan H (2009). Bloodstream infections caused by ESBL-producing E. Coli and K. Pneumoniae: risk factors for multidrug-resistance. Braz J Infect Dis.

[24] Hansen F, Olsen SS, Heltberg O, Justesen US, Fuglsang-Damgaard D, Knudsen JD (2014). Characterization of third-generation cephalosporin-resistant *Escherichia coli* from bloodstream infections in Denmark. Microbial drug resistance (Larchmont, NY).

[25] Courpon-Claudinon A, Lefort A, Panhard X, Clermont O, Dornic Q, Fantin B (2011). Bacteraemia caused by third-generation cephalosporin-resistant Escherichia coli in France: prevalence, molecular epidemiology and clinical features. Clinical microbiology and infection : the official publication of the European Society of Clinical Microbiology and Infectious Diseases.

[26] Pobiega M, Wojkowska-Mach J, Chmielarczyk A, Romaniszyn D, Adamski P, Heczko PB (2013). Molecular characterization and drug resistance of Escherichia coli strains isolated from urine from long-term care facility residents in Cracow, Poland. Medical science monitor : international medical journal of experimental and clinical research.

[27] Lautenbach E, Han J, Santana E, Tolomeo P, Bilker WB, Maslow J (2012). Colonization with extended-spectrum beta-lactamase-producing Escherichia coli and Klebsiella species in long-term care facility residents. Infect Control Hosp Epidemiol.

[28] Ku NS, Kim YC, Kim MH, Song JE, Oh DH, Ahn JY (2014). Risk factors for 28-day mortality in elderly patients with extended-spectrum beta-lactamase (ESBL)-producing Escherichia coli and Klebsiella pneumoniae bacteremia. Arch Gerontol Geriatr.

[29] Yang YS, Ku CH, Lin JC, Shang ST, Chiu CH, Yeh KM (2010). Impact of extended-spectrum beta-lactamase-producing Escherichia coli and Klebsiella pneumoniae on the outcome of community-onset bacteremic urinary tract infections. Journal of microbiology, immunology, and infection =. Wei mian yu gan ran za zhi.

[30] Kang CI, Wi YM, Ko KS, Chung DR, Peck KR, Lee NY (2013). Outcomes and risk factors for mortality in community-onset bacteremia caused by extended-spectrum beta-lactamase-producing Escherichia coli, with a special emphasis on antimicrobial therapy. Scand J Infect Dis.

[31] Peralta G, Lamelo M, Alvarez-Garcia P, Velasco M, Delgado A, Horcajada JP (2012). Impact of empirical treatment in extended-spectrum beta-lactamase-producing Escherichia coli and Klebsiella spp bacteremia A multicentric cohort study. BMC Infect Dis.

[32] Joo EJ, Park DA, Lee NR, Moon SY, Choi JK, Ko JH (2017). Impact of appropriateness of empiric therapy on outcomes in community-onset bacteremia by extended-spectrum-beta-lactamase producing Escherichia coli and Klebisella pneumoniae definitively treated with carbapenems. European journal of clinical microbiology & infectious diseases : official publication of the European Society of Clinical Microbiology.

[33] Joo EJ, Park DA, Lee NR, Moon SY, Choi JK, Ko JH, et al. Impact of appropriateness of empiric therapy on outcomes in community-onset bacteremia by extended-spectrum-beta-lactamase producing Escherichia coli and Klebisella pneumoniae definitively treated with carbapenems. European journal of clinical microbiology & infectious diseases : official publication of the European Society of Clinical Microbiology. 2017. 10.1007/s10096-017-3031-7 28643188

[34] Cheng WL, Hsueh PR, Lee CC, Li CW, Li MJ, Chang CM (2016). Bacteremic pneumonia caused by extended-spectrum beta-lactamase-producing *Escherichia coli* and *Klebsiella pneumoniae*: Appropriateness of empirical treatment matters. J Microbiology, immunology, and infection = Wei mian yu gan ran za zhi.

[35] Heng ST, Chen SL, Wong JGX, Lye DC, Ng TM (2018). No association between resistance mutations, empiric antibiotic, and mortality in ceftriaxone-resistant Escherichia coli and Klebsiella pneumoniae bacteremia. Sci Rep.

[36] de Kraker ME, Wolkewitz M, Davey PG, Koller W, Berger J, Nagler J (2011). Burden of antimicrobial resistance in European hospitals: excess mortality and length of hospital stay associated with bloodstream infections due to Escherichia coli resistant to third-generation cephalosporins. J Antimicrob Chemother.

[37] Tschudin-Sutter S, Frei R, Stephan R, Hachler H, Nogarth D, Widmer AF (2014). Extended-spectrum beta-lactamase (ESBL)-producing Enterobacteriaceae: a threat from the kitchen. Infect Control Hosp Epidemiol.

